# Comparison of Visible Light‐Protective Tinted Sunscreen to Untinted Sunscreen to Protect Melasma Patients During Summer: A Prospective Randomized Investigator‐Blinded Study

**DOI:** 10.1111/jocd.70450

**Published:** 2025-09-27

**Authors:** Helena Polena, Catherine Queille‐Roussel, Christelle Graizeau, Luc Duteil, Michèle Sayag, Thierry Passeron

**Affiliations:** ^1^ Research and Development Department NAOS Ecobiology Company (Bioderma) Aix‐en‐Provence France; ^2^ Center of Clinical Pharmacology Applied to Dermatology (CPCAD) L'Archet 2 Hospital Nice France; ^3^ Institut NAOS des Sciences de la Vie Aix‐en‐Provence France; ^4^ Department of Dermatology, Centre Hospitalier Universitaire de Nice University Côte d'Azur Nice France; ^5^ C3M, INSERM U1065, University Côte d'Azur Nice France

**Keywords:** melasma, photoprotection, skin pigmentation, tinted sunscreen, visible light

To the Editor,

Melasma is a common hyperpigmentation skin disorder characterized by relapses due to sun exposure. While ultraviolet (UV) radiation is the main cause of skin pigmentation, recent studies highlighted the significant role of visible light (VL, 400–700 nm), especially in melasma relapses [1]. Broad‐spectrum sunscreens protect against UV radiation but offer limited VL protection. In contrast, tinted sunscreen containing iron oxides and pigmentary titanium dioxide effectively reduces VL transmittance [2]. Therefore, sunscreens against UVA, UVB, and VL are key in melasma prevention [3]. However, clinical studies comparing tinted and untinted sunscreens in melasma patients remain scarce.

This study aimed to compare melasma relapse prevention by a VL‐protective tinted sunscreen versus an untinted sunscreen (see Supporting Information for compositions S1). A single‐center, randomized, investigator‐blinded, and controlled clinical study was conducted in Southern France during summer. The tinted sunscreen (Photoderm M SPF50+ Golden tinted, Bioderma, France) contains VL filters (iron oxides and pigmentary titanium dioxide, pVL protection factor 66) [4] and UV filters (UVA‐PF = 35, SPF = 65). The untinted sunscreen had a similar composition and protection against UV radiation (UVA‐PF = 36, SPF = 64), but no dedicated VL filters.

Forty‐two women with melasma (aged: 28–58, mean = 39.5) and Phototype III (93%) or IV (7%) were recruited (study design, ethics, assessments and statistical analysis are detailed in the supporting Information S1). Half of them, randomly selected, applied the tinted sunscreen at least twice daily during 5 months, while the other half applied the untinted sunscreen. The primary endpoint was the objective assessment of pigmentation using colorimetric analysis. Skin pigmentation was evaluated using the L* (lightness), a* (redness), and b* (yellowness) parameters, measured with the Konica‐Minolta Chromameter CR400, focusing on a melasma‐affected facial region and an adjacent unaffected area. The Individual Typological Angle (ITA°), inversely related to pigmentation, was calculated from the L* and b* values. The ΔE color homogeneity parameter, representing the color difference between lesional and perilesional skin, was derived from the L*a*b* values of the two areas. Besides, melasma severity was clinically assessed by two physicians using the modified Melasma Area and Severity Index (mMASI) [5]. All evaluations were performed before cream application, at baseline, after 2.5 and 5 months (T2.5 and T5 months) with three measurements taken per area and timepoint. Product tolerance was monitored throughout the study.

Of 42 participants, 41 completed the study, one being withdrawn due to pregnancy. Both sunscreens were well tolerated, with no significant difference in usage between groups. Despite significant differences, or differences at the limit of significance, at baseline between the untinted and the tinted groups, the mean L* and ITA° values remained stable or slightly increased for both areas and groups, indicating that both sunscreens prevented sunlight‐induced skin darkening and pigmentation. After 5 months, the mean difference between melasma and unaffected areas for L*, ITA°, and ΔE (∆L*, ∆ITA°, and ∆E) was significantly reduced in the tinted sunscreen group but not in the untinted group (Figure 1). Differences in lightness (∆L*), pigmentation (∆ITA°), and color contrast (∆E) were significantly lower after 5 months of tinted sunscreen use. No significant changes were observed for a* and b* parameters (data not shown). The mMASI significantly decreased after 5 months with both tinted (3.5 vs. 4.0, *p* < 0.001) and untinted (3.2 vs. 4.1, *p* < 0.001) sunscreens, without significant difference between them. Clinical examples are provided in Figure 2.

**FIGURE 1:**
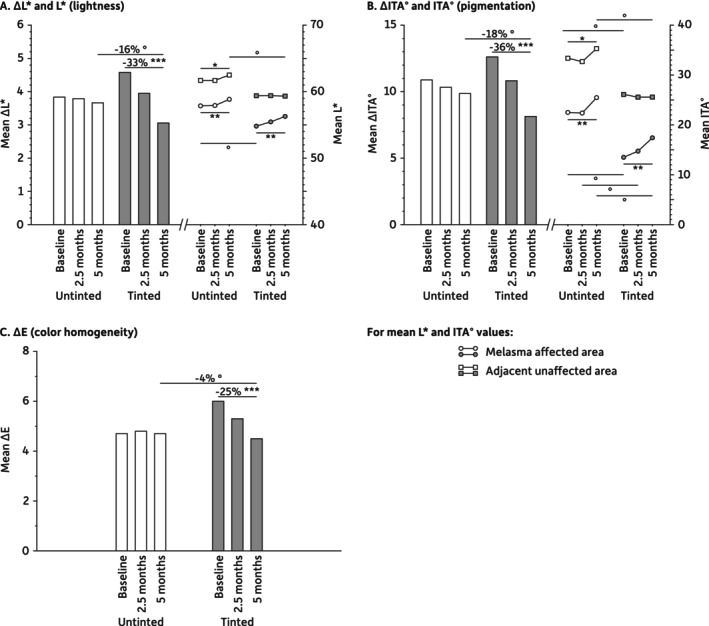
Evolution of (a) ∆L* and L* (lightness), (b) ∆ITA° and ITA° (pigmentation), and (c) ∆E (color homogeneity) parameters. For all parameters, variations (∆) between the melasma affected and unaffected areas in the untinted and tinted groups are presented as histograms. Mean parameter values (L* and ITA°) for the melasma affected and unaffected areas in the untinted and tinted groups are presented as dots and lines. Inter‐group differences (°) were assessed using unpaired t‐test at identical timepoints. Intra‐group differences (*) versus baseline were analyzed using Dunnett's adjustments for variations (∆) and paired t‐test for L* and ITA°. Statistical differences are reported with * or °: *p* < 0.05, **: *p* < 0.01, and ***: *p* < 0.001.

**FIGURE 2:**
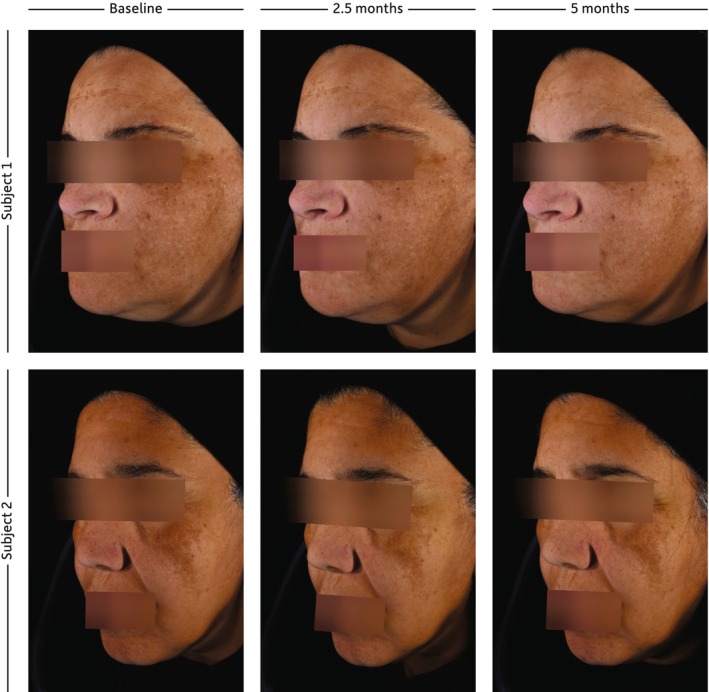
Photographs of two subjects who applied the untinted or the tinted sunscreen for 5 months.

Despite being conducted in summer, this study objectively demonstrates that the daily use of a high protection UVB/UVA sunscreen prevents the recurrence of melasma, as evidenced by the stability and even the lightening of hyperpigmented areas of skin. Interestingly, the addition of VL‐protective pigments to the sunscreen further improved the uniformity of pigmentation between the melasma‐affected skin and the non‐affected skin significantly. These findings support international expert recommendations for the year‐round use of broad‐spectrum tinted sunscreens in melasma patients [6].

## Author Contributions

**Luc Duteil and Catherine Queille‐Roussel:** performed the research and Helena Polena. **Michèle Sayag and Thierry Passeron:** assisted with editorial content. **Helena Polena, Michèle Sayag and Thierry Passeron** designed the study. **Helena Polena, Christelle Graizeau, Michèle Sayag and Thierry Passeron** supervised the study. All authors reviewed, read, and approved the final manuscript.

## Conflicts of Interest

H.P., C.G., and M.S. are or were employees of NAOS. T.P. has received honoraria from Beiersdorf, Galderma, L'Oréal, Hyphens, ISIS Pharma, ISDIN, NAOS, Pierre Fabre, SUN Pharma, SVR, and Symrise.

## Supporting information


**Data S1:** Supporting Information.

## Data Availability

The data that support the findings of this study are available from the corresponding author upon reasonable request.
