# Supplementary material for: Comparison of Visible Light‐Protective Tinted Sunscreen to Untinted Sunscreen to Protect Melasma Patients During Summer: A Prospective Randomized Investigator‐Blinded Study
Source: J Cosmet Dermatol. 2025 Sep 27;24(10):e70450. doi: 10.1111/jocd.70450 (PMC12475913; doi:10.1111/jocd.70450)
Supplement: Supplementary file 1 — Data S1: Supporting Information. [file JOCD-24-e70450-s001.docx]

**Supporting information – Detailed Material and Methods**

- 1. **Investigated products**

One of the investigated product is a commercially available visible light-protected tinted sunscreen (Photoderm M SPF50+ Golden tinted, Bioderma, France). The second product is the same sunscreen formulation deprived of visible light protection.

Both products share the following composition: AQUA/WATER/EAU, DIBUTYL ADIPATE, TITANIUM DIOXIDE [NANO], DIISOPROPYL SEBACATE, DIETHYLAMINO HYDROXYBENZOYL HEXYL BENZOATE, METHYLENE BIS-BENZOTRIAZOLYL TETRAMETHYLBUTYLPHENOL [NANO], DIETHYLHEXYL BUTAMIDO TRIAZONE, POTASSIUM CETYL PHOSPHATE, BIS-ETHYLHEXYLOXYPHENOL METHOXYPHENYL TRIAZINE, CORN STARCH MODIFIED, PENTYLENE GLYCOL, PROPANEDIOL, SILICA, C20-22 ALKYL PHOSPHATE, C20-22 ALCOHOLS, DECYL GLUCOSIDE, TOCOPHERYL ACETATE, POLYACRYLATE CROSSPOLYMER-6, CAPRYLYL GLYCOL, XANTHAN GUM, ECTOIN, MANNITOL, XYLITOL, RHAMNOSE, PROPYLENE GLYCOL, SODIUM HYDROXIDE, FRUCTOOLIGOSACCHARIDES, CAPRYLIC/CAPRIC TRIGLYCERIDE, and LAMINARIA OCHROLEUCA EXTRACT [BI 711].

To ensure visible light protection, the tinted product additionally contains: TITANIUM DIOXIDE (CI 77891), and IRON OXIDES (CI 77492, CI 77491, CI 77499). It also contains SODIUM LAUROYL GLUTAMATE, LYSINE, MAGNESIUM CHLORIDE, and GLABRIDIN that were not present in the untinted sunscreen.

The UV protection of both products was evaluated according to ISO 24442:2022 and ISO 24444:2019 standards by an independent laboratory (Helioscience, France). The visible light protection was assessed according to Duteil *et al*. [4]. Based on these assessments, the tinted sunscreen demonstrated a protection against visible light (pVL) of 66, with UVA-PF = 35 and SPF = 65. The untinted sunscreen showed a UVA-PF = 36 and SPF = 64. Deprived of specific visible light protective ingredients, its pVL protection factor was not evaluated.

- 1. **Study design**

The study was a single center, randomized, investigator-blinded, and controlled clinical study designed to evaluate the efficacy of the tinted sunscreen *versus* that of the untinted sunscreen in protecting melasma patients. Conducted under dermatological supervision in Southern France during the summer, the study aimed to determine whether a sunscreen offering visible light protection was more effective than one with comparable UVA and UVB protection but reduced visible light protection.

The study enrolled 42 participants (male or female, aged 28–58 years, mean age = 39.5, Fitzpatrick skin types III–IV), all diagnosed with melasma. Subjects were randomly assigned, prior to any assessments, to one of two parallel groups (n = 21 each) using either the tinted or the untinted sunscreen. Participants applied the assigned product to the entire face twice daily (morning and afternoon). Additional applications were possible every two hours during sun exposure, after sweating, swimming, or towel drying.

- 1. **Ethics**

The study protocol was approved by the *Comité de Protection des Personnes Est I* on April 5, 2022. The study complied with the Declaration of Helsinki, Good Clinical Practice (GCP), and local regulations. All participants provided written informed consent after being fully informed about the study’s purpose, procedures, and potential risks.

- 1. **Instrumental assessments**

Assessments were performed at baseline (day 1), after 2.5 months, and at the study completion (5 months). At each time point, colorimetric measurements (L*, a*, b*) were taken using a Konica-Minolta Chromameter^®^ CR400 on a melasma-affected facial area and an adjacent unaffected area. Analyses focused on:

- The L* parameter, representing skin lightness
- The ITA° angle, representing the pigmentation intensity, calculated as:

ITA° = [Arc tan ((L* - 50)/b*)] x 180/π

- The ∆E color difference between melasma-affected and unaffected areas, according to the formula:

$$\text{∆E = }\sqrt{\text{∆L*}^{\text{2}}\text{ + }\text{∆a*}^{\text{2}}\text{ + }\text{∆b*}^{\text{2}}}$$

- 1. **Statistical analysis**

Results are presented as the mean ± standard deviation (SD). Data distribution was evaluated using the Shapiro-Wilk test (α<0.1). Normally distributed, mean L* and ITA° values were assessed using paired t-test *versus* baseline for intra-group analysis and unpaired t-test between identical time points for inter-group analysis. Unpaired t-test were also used to evaluate inter-group variations (∆L*, ∆ITA°, and ∆E) at identical time points. Intra-group analysis relied on Dunnett’s adjustments.
